# The comparison of endotracheal intubation and laryngeal tube insertion with face-to-face method in in-vehicle traffic accidents

**DOI:** 10.1007/s11739-025-03885-8

**Published:** 2025-02-17

**Authors:** Merve Arslan, Ali Ekşi

**Affiliations:** 1 Institute of Health Sciences Disaster Medicine Department, Ege University, Izmir, Turkey; 2 Atatürk Health Care Vocational School, Ege University, Bornova, 35100 Izmir, Turkey

**Keywords:** Endotracheal intubation, Pre-hospital emergency Care, Advanced airway applications, Laryngeal tube, Supraglottic airway methods, Face to face airway applications

## Abstract

Emergency airway management is a critical focus in prehospital emergency healthcare. The right technique and the right equipment may increase survival. The study aimed to compare endotracheal intubation and laryngeal tube insertion with the face-to-face method in difficult conditions such as in-vehicle traffic accidents in which the injured person is trapped inside the vehicle. The population of the study, which was carried out as experimental research, consisted of emergency health workers currently working in Bursa 112 Ambulance Services (*n*: 383). The study compared two different airway applications with face-to-face techniques using a simulator mannequin. Data were collected between February and May 2023 and the IBM Statistical Package for Social Sciences for Windows (SPSS 25) computer program was used for statistical data analysis. The suitability of the numerical variables for normal distribution was tested by the Shapiro–Wilk test. Since the variables did not conform to the normal distribution, they were given as median (Q1–Q3) values. Participants’ preparation, implementation, and total times for ETI and LT were compared using the Wilcoxon test. The duration of face-to-face ETI and LT times were compared regarding participants' personal characteristics, experience status, and the training they received with the Mann–Whitney U test and the Kruskal–Wallis test. Categorical variables are given as number and percentage values. *p* < 0.05 was considered significant. In face-to-face endotracheal intubation, 24.3% of the participants were successful in the first attempt, 30% in the second attempt, 27.1% in the third attempt, 18.6% failed in all three attempts, and 38.6% performed esophageal intubation. 87.1% of the participants were successful in face-to-face laryngeal tube insertion in the first and 12.9% in the second attempt. The duration of face-to-face laryngeal tube placement was found to be significantly shorter than the duration of endotracheal intubation (*p* < 0.05). In face-to-face airway conduct, the length of endotracheal intubation time and the high risk of esophageal intubation make the laryngeal tube more advantageous than endotracheal intubation. Furthermore, the high number of attempts required for successful face-to-face endotracheal intubation may pose additional risks by causing destabilization in trauma patients requiring cervical stabilization.

## Introduction

Traffic accidents are already an important public health problem, especially in developing countries [1], and most deaths in traffic accidents occur at the prehospital emergency health services (PH-EHS) stage [2]. Airway conduct has an important place in the PH-EHS which is enabled in traffic accidents, and early airway control and protection of the patient from hypoxia are directly related to mortality [3]. In patients who have had an in-vehicle traffic accident and are trapped in the vehicle, airway conduct may be required until the patient is rescued, and face-to-face treatments may become a preference as the most ideal mode of transport for the patient [4, 5]. In “face-to-face” treatments, also known as the “tomahawk” or “pickaxe” method, one person manually stabilizes the head and neck in a neutral position, while the other person holds the handle of the laryngoscope in the right hand and places the plate on the upper part of the tongue, in contrast to conventional laryngoscopy [6]. In face-to-face endotracheal intubation (ETI), contrary to the standard method, normal laryngoscopy is not possible due to limited access to the patient’s head, the treatment time may be prolonged, and more complications may be encountered [7].

Although supraglottic airway tools (SGATs), both as the primary tool and as an alternative in case of ETI failure, have shown remarkable development in the last 20 years [8–10], the pros and cons of SGATs compared to ETI are still debated. In particular, the success and efficiency of laryngeal tube (LT) treatment attracts attention and is considered an important option in cases where access to the patient is limited and after failed ETI [11, 12]. In the study of Van Tulder et al. (2020), it was reported that the use of LT has increased significantly in recent years compared to ETI in PH-EHS [13]. Although the area of use of LT has expanded in the PH-EHS [14, 15], there is a need for further research on the use of the tool in different conditions and environments [16].

Although face-to-face method airway conduct has been recommended in many studies [15, 17, 18] as a feasible method in PH-EHS, there is insufficient evidence, especially for face-to-face method LT treatment. Although there are many studies in the literature comparing standard (supine) position treatments of airway tools and comparing ETI with various SGATs in-vehicle traffic accidents, there are not enough studies comparing face-to-face ETI with LT treatments. In this study, we aimed to compare ETI and LT with the face-to-face method. In this way, it is aimed to contribute to the discussions on airway management in PH-EHS, where there are many areas on which there is no complete agreement.

## Methods

### Design

The study was planned as experimental research. The study compared two different airway treatments with face-to-face techniques using a simulator mannequin. ETI and King-LT were used among airway methods. Cuffed endotracheal tube number 6.5 and King-LT number 4 were used due to their compatibility with the simulator mannequin.

### Place and time of the research

The research was conducted at the Bursa Provincial Health Directorate Simulation Training, Research, and Application Centre between February and May 2023.

### Participants

The population of the study consisted of employees working currently in Bursa 112 Ambulance Services (*n*: 383). The sample size for intervention research: the significance of the difference between two dependent groups was calculated as 70 people with a *t*-test distribution (matched pairs), an effect size (w) of 0.25, 80% power, and a one-way test at a significance level of 0.05 (α) via using the G-Power statistical program. With the prediction that volunteers who were excluded from the study and who dropped out could create a deviation of 20%, 84 people were invited to the study. All participants are individuals who have completed their airway management training using the standard method, including in-service training provided by the workplace in addition to their vocational school training. The volunteers were warned that they could leave the study at any stage of the study.

### Data collection

**Figure 1:**
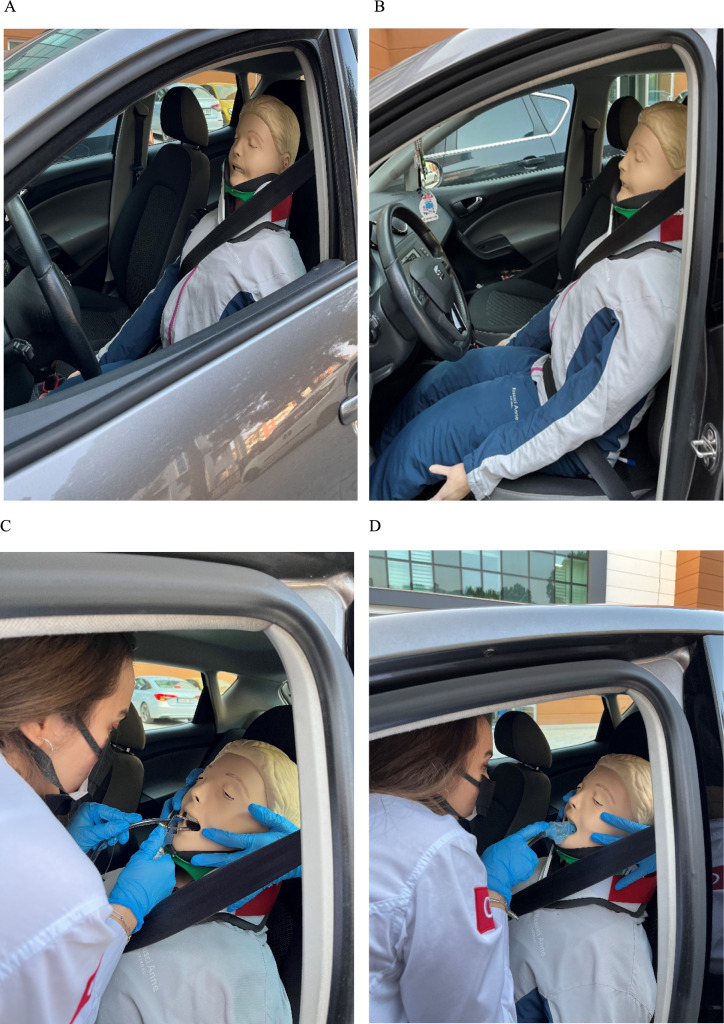
In-Vehicle Position of Simulator Mannequin and Face-to-Face ETI-LT Treatments

First, verbal and written informed consent was obtained from the participants and then their characteristics (age, gender, educational status, and professional experience) were recorded on the case form. A simulator mannequin (QCPR®) that can digitally describe successful airway treatment was used for data collection. The simulator mannequin and the materials used during the treatment were introduced to the participants in detail. In addition, the participants were informed about the face-to-face airway technique and they were allowed to perform as many ETI and LT treatment experiments as they wanted on the simulator mannequin. The mannequin with cervical collar was placed on the driver’s seat of a car (Seat Ibiza® 2016 Model), the seat belt was attached to the body of the manikin and the seat was moved as far forward as possible to simulate entrapment (Figure 1).

The participants were told that there was a patient who was unconscious and in need of emergency airway conduct and that they should perform airway conduct with the face-to-face method. The participants were asked to perform face-to-face ETI first. After the participant performed the ETI treatment, a break was given for rest, and after the break, he was asked to perform the LT treatment under the same conditions. The practitioner was assisted by only one person throughout the procedure, who provided only cervical immobilization (Figure 1).

## Measurement of insertion/treatment time

During the treatment, an emergency aid bag containing all the necessary materials for the treatment was kept ready next to the practitioner. In both ETI and LT treatments, a stopwatch was started as soon as the practitioner touched the bag and the time was stopped when a “normal chest rise (ventilation time)” warning was received from the simulator mannequin. The procedure duration was calculated in three ways: time during preparation, time during tube placement, and total time. In contrast, preparation time was defined as the time required for the operator to prepare all materials and equipment necessary for the application, and tube placement time was defined as the time needed for the airway device to be inserted and fixed into the airway through the mouth. The total time defined the entire preparation and tube placement time. For ETI, esophageal intubation, exceeding the given time, failure to raise the chest with ventilation, and failure of the participant to succeed in three attempts were considered unsuccessful attempts, but removing and reinserting the laryngoscope was not regarded as unsuccessful attempts unless the time was exceeded. On the other hand, for LT, failure to complete the attempt within the given time and to raise the chest with ventilation were considered unsuccessful. For those who failed the procedure, the procedure was restarted and the time was measured again. Esophageal intubation was considered an unsuccessful attempt. The participants were given a maximum of three attempts in both treatments. The participants were blinded to the study results measured and recorded during the simulation.

### Data analysis and evaluation techniques

IBM Statistical Package for Social Sciences for Windows (SPSS 25) computer program was used for statistical analysis of the data. The suitability of the numerical variables for normal distribution was tested by the Shapiro–Wilk test. Since the variables did not conform to the normal distribution they were given as median (Q1–Q3) values. Participants’ preparation, implementation, and total times for ETI and LT were compared using the Wilcoxon test. The duration of face-to-face ETI and LT times were compared in terms of participant's personal characteristics, experience status, and the training they received with the Mann–Whitney U test and Kruskal–Wallis test. The categorical variables are given as number and percentage values. *p* < 0.05 was considered significant.

### Ethics of the study

Ethics Committee Approval dated 18.11.2021 and numbered 21–11.1 T/30 was obtained from Xx University Medical Research Ethics Committee.

## Results

### Participant descriptive findings

The study was completed with 70 participants after those who were excluded from the study and those who dropped out of the study. The mean age of the participants was 27.06 ± 4.98 years, the median age was 25 years (minimum–maximum: 21–40), 74.3% (*n* = 52) were in the age range of 20–29 years, 61.4% (*n* = 43) were women, and 68.6% (*n* = 48) had 1–5 years of professional experience (Table 1).

**Table 1:** Personal characteristics of the participants

Variables	*n*	%
Age		
20–29	52	74.3
30–39	17	24.3
40 and over	1	1.4
Gender		
Female	43	61.4
Male	27	38.6
Professional experience		
1–5	48	68.6
6–10	12	17.1
11–15	6	8.6
16–20	3	4.3
21 and over	1	1.4

All the participants received standard method (supine) practical training on ETI and LT treatments. Face-to-face method airway conduct applied training was “yes” in 5.7% (*n* = 4) and “no” in 94.3% (*n* = 66). None of the participants had ever performed ETI or LT with the face-to-face method in a real patient.

All participants except one (1.4%) had experience with standard method ETI application, while 4 (5.7%) had experience with standard method LT application. None of the participants had experience with face-to-face ETI or LT application (Table 2).

**Table 2:** Participants’ previous experience with ETI -LT

Variables	*n*	%
Previous experience with standard method ETI		
Never	1	1.4
1–5 times	23	32.9
6–10	15	21.4
11–20	6	8.6
21 and over	25	35.7
Previous experience with face-to-face ETI method		
Never	70	100.0
Previous experience with standard method LT		
Never	66	94.3
1–5 times	4	5.7
6–10	0	0.0
11–20	0	0.0
21 and over	0	0.0
Previous experience with face-to-face LT method		
Never	70	100.0

When the number of attempts for successful treatments of face-to-face ETI was evaluated; 24.3% (*n* = 17) were successful in the first attempt, 30.0% (*n* = 21) in the second attempt, 27.1% (*n* = 18) in the third attempt, and 18.6% (*n* = 13) failed. 48.7% of the participants performed esophageal intubation in the first attempt. In the number of successful LT treatment attempts with the face-to-face method, 87.1% (*n* = 61) were successful in the first attempt, 12.9% (*n* = 9) were successful in the second attempt, and there were no unsuccessful participants (Table 3).

**Table 3:** First pass success for face-to-face method ETI and LT

Number of experiments for successful treatments of face-to-face method	ETI		LT	
Variables	*n*	%	*n*	%
First Experiment	17	24.3	61	87.1
Second Experiment	21	30.0	9	12.9
Third Experiment	19	27.1	0	0.0
Failed	13	18.6	0	0.0

### Comparison of face-to-face method ETI and LT treatment times

ETI preparation median time was 45.50 (Q1–Q3: 37.00–56.25) s, ETI tube placement median time was 47.00 (Q1–Q3: 30.00–60.25) s, and ETI total median time was 96.00 (Q1–Q3: 77.75–108.25) s. LT preparation median time was 25.00 (Q1–Q3: 20.75–31.25) s, LT tube placement median time was 19.00 (Q1–Q3: 14.00–25.00) s, LT total median time was 44.00 (Q1–Q3: 38.50–55.25) s (Table 4).

**Table 4:** Comparison of face-to-face method ETI-LT treatment times

Time	ETI (*n* = 70)	LT (*n* = 70)	*p* value
Preparation	45.50 (37.00–56.25)	25.00 (20.75–31.25)	< 0.001
Tube Placement	47 (30.00–60.25)	19.00 (14.00–25.00)	< 0.001
Total	96.00 (77.75–108.25)	44.00 (38.50–55.25)	< 0.001

^*^Data presented as median (Q1–Q3) values

A statistically significant difference was found in the face-to-face method ETI -LT treatment times comparison (*p* < 0.001). LT preparation time, LT tube placement time, and LT total time were shorter than ETI preparation time, ETI tube placement time, and ETI total time (Table 4).

There was no statistically significant difference between the participant’s personal characteristics, experience status, the training they received, and the duration of face-to-face ETI and LT treatment (*p* < 0.05).

## Discussion

There are few high-quality studies on effective, safe, and optimal airway management techniques in presenting PH-EHS [19, 20]. When the literature is examined, there are not enough studies to decide on the correct method for airway applications in PH-EHS, comparing application times between methods and evaluating skill status [8, 9]. Various SGATs have been compared within themselves but no model studies comparing face-to-face ETI with LT were found. Despite positive indications of the efficacy and safety of SGATs in the literature, the lack of specific research (especially randomized controlled trials) in PH-EHS complicates the decision-making process regarding best practices in PH-EHS airway management [10]. However, the pros and cons of SGATs compared to ETI are still under debate, and there are not enough studies, especially in the context of investigating the success rates and effectiveness of LT from alternative airway equipment [14, 16]. In this study, it is seen that the findings regarding the effectiveness and duration of LT application are promising in situations with limited patient-injured access such as in-vehicle traffic accidents.

In this study, most of the participants did not receive practical training on face-to-face ETI and face-to-face LT, and none of them had experience in applying face-to-face ETI and face-to-face LT. However, it is seen that the success rate in the first attempt in the face-to-face LT application is approximately four times higher than in the face-to-face ETI application. When the training curricula of PH-EHS employees are evaluated, it is seen that the standard method, which is planned with the patient in the supine position and the health professional at the head of the stretcher, is preferred in the application training related to airway management [21–23]. The reasons such as ease of use due to their simple design, high success rates, ventilation like ETI [24], not requiring much experience, and being able to be applied quickly indicate that SGATs, including LT, can be used more widely in PH-EHS [8]. In addition, it is supported by the literature that the skills related to SGATs can be developed much faster thanks to the training to be given to PH-EHS employees [25]. LT application success rates are reported as 72–94% in PH-EHS employees with short training using training models [15, 26, 27]. In cases where there are various difficulties in terms of airway management, especially in vehicle traffic accidents, training plans related to LT application in the face-to-face method can be reviewed again.

It is known that ETI performed by prehospital emergency healthcare workers is associated with more negative outcomes compared to the in-hospital field. These negative outcomes may be due to many factors, including environmental factors such as inadequate lighting or narrow scene environment, patient factors, and practitioner experience [28]. If the PH-EHS worker encounters ETI treatment infrequently and exposure is rare, the failure of treatment can be up to 50% and treatment requires many repeated efforts [29]. Although it was emphasized in the study of Venezia et al. (2012) that the success rate was higher in face-to-face ETI treatments compared to standard ETI treatment, the success rate of face-to-face ETI was also found to be quite low in this study [22]. In this study, only approximately one-fourth of the participants were successful in face-to-face ETI in the first attempt. Such high failure rates necessitate the discussion of the use of alternative airway tools to ETI in face-to-face airway conduct.

In the study of Benedetto et al. (2007), the rate of esophageal intubation in emergency ETI treatments in the hospital was given as 9%, and the rate may increase up to 30% in PH-EHS [22, 30]. The studies have indicated that an unidentified esophageal intubation rate of more than 10% in emergency cases including cardiac arrests is not acceptable [12, 31]. In this study, approximately half of the participants performed esophageal intubation on the first attempt in ETI using a face-to-face technique. The fact that the rate of esophageal intubation in face-to-face ETI treatment is considerably higher than the rates of esophageal intubation in standard ETI treatment reflected in the literature is important evidence to discuss the use of other tools instead of ETI.

The superiorities of SGATs over ETI, such as easier use [10] and shorter treatment time [30], are discussed in the literature. With the effect of studies demonstrating the adequacy of SGATs in terms of airway safety and efficiency [32], some PH-EHS systems emphasize alternative airway tools, especially LT, in airway conduct [33]. There are studies in the literature [30] showing that especially inexperienced healthcare workers use SGATs more easily compared to ETI. Some studies prioritize LT over ETI in terms of speed of treatment [34]. Again, in many studies performed on simulator manikins [24, 35], the speed, efficacy, and safety of LT with standard treatment have been studied. However, there are not enough studies that can provide evidence for face-to-face LT treatment in PH-EHS. The data from this study provide evidence that LT can be used in face-to-face airway management in terms of both effectiveness and duration of application.

In peri-arrest conditions and cases such as airway obstruction, urgent advanced airway conduct may be required [36]. Pap et al. (2019) reported in their in-vehicle traffic accident simulation study that SGATs provide advantages in terms of time and ease of use compared to ETI [37]. Steinmann et al. (2016) stated in their study that SGATs can be used as alternative airway tools in in-vehicle traffic accidents after ETI failure and emphasized that SGATs provide half the time savings compared to ETI [38]. Martin et al. (2016) reported that LT provided a significant time advantage over ETI in their in-vehicle traffic accident simulation study [39]. In this study, the preparation, treatment, and total times related to ETI were approximately two times longer than LT. Although the time advantage is important in terms of rapid initiation of ventilation to the patient, it is also important in terms of providing more time for other life-saving interventions to be performed in PH-EHS.

In the mannequin studies in the literature [36–39] in which the injured person trapped in the vehicle was simulated, no clear method was suggested. This may be because the wounded may be in many different positions in the vehicle in traffic accidents. However, it has been shown that in in-vehicle traffic accidents, if the seat belt is fastened, the patient remains in a sitting position in the vehicle seat [40, 41]. This situation makes it necessary to recommend more clearly that airway conduct can be performed with face-to-face technique under appropriate conditions in vehicle traffic accidents. In the study of Grosomanidis et al. (2012), it was stated that face-to-face methods and ETI treatment could be used in PH-EHS [17]. However, since normal laryngoscopy is not possible in face-to-face ETI treatment, the treatment time may be prolonged [7, 42]. In the study of Julliard et al. (2022), the face-to-face method ETI treatment time was found to be longer than the standard method ETI treatment time [43]. Considering the relationship between trauma-related mortality rates and the duration of time spent in the PH-EHS [44], it can be said that face-to-face LT treatment, which provides half the time advantage in the data of this study, provides more advantage than face-to-face ETI treatment in-vehicle traffic accidents.

One of the most important injuries in vehicle traffic accidents is spinal trauma and airway conduct should be performed with cervical immobilization, especially in patients with suspected cervical injury [45]. In patients with cervical immobilization, it becomes difficult to see the vocal cords during laryngoscopy [31] and laryngoscopy performed during face-to-face ETI also makes cervical immobilization difficult [46]. In addition, repeated and prolonged laryngoscopy procedures for successful treatment in face-to-face ETI may increase morbidity and mortality [29]. Therefore, while providing airway conducts in-vehicle traffic accidents, although time is of course an important variable, the number of attempts for successful airway treatment also becomes an important factor. In non-standard ETI treatments such as the face-to-face method, the risk of misplacing the tube increases with the prolongation of the time spent for airway conduct [44], which increases the number of attempts for successful treatment. In this study, only approximately one-quarter of the participants were successful in the first attempt in face-to-face ETI, whereas the success rate in the first attempt in face-to-face LT was around 90% in parallel with the literature [15]. LT provides a higher success rate with its ability to be applied more rapidly compared to ETI [9]. This is very important in terms of cervical stabilization in traumatized patients.

## Conclusion

Face-to-face airway conduct can be used in various situations where access to the patient is restricted, particularly in-vehicle traffic accidents. In face-to-face airway conduct, the length of ETI treatment time and the high risk of esophageal intubation make LT more advantageous than ETI. In addition, the high number of attempts for successful treatment in face-to-face ETI may pose additional risks by causing destabilization in trauma patients requiring cervical stabilization.

## Statements and declarations

The authors report that there are no competing interests to declare. There is no financial support related to this work.

## Limitations

First, this study performed a traffic accident simulation with a simulator mannequin and a car. Therefore, the data of this study may not fully reflect the actual patient and event data. In vehicle traffic accidents, advanced airway management may not be required for many patients. In many patients, adequate oxygenation can be provided with oropharyngeal or nasopharyngeal airway + bag-mask ventilation by using the face-to-face method. However, in cases where hypoxia occurs due to various reasons, especially chest trauma and long-term rescue operations, advanced airway management with the face-to-face method can be life-saving.
